# An Investigation on the Ball Screw-Based Variable Displacement Mechanism for Axial Piston Pumps with Feedforward Differential Input Control

**DOI:** 10.3390/s25040994

**Published:** 2025-02-07

**Authors:** Guangcheng Zhang, Bokai Wang, Yueh-Jaw Lin

**Affiliations:** 1 School of Mechanical Engineering, University of Shanghai for Science and Technology, Shanghai 200093, China; g.c.zhang@usst.edu.cn (G.Z.); 233381540@st.usst.edu.cn (B.W.); 2 College of Engineering and Engineering Technology, Northern Illinois University, DeKalb, IL 60115, USA

**Keywords:** axial piston pump, displacement control, pump-controlled system, feed-forward compensation

## Abstract

This paper proposes a variable mechanism structure based on a ball screw design for precise displacement control in axial piston pumps, with the objective of improving actuator position and velocity control within the displacement-controlled (DC) systems. Traditional valve-controlled cylinder variable mechanisms (VCCVM) often suffer from limited control precision over the swash plate due to numerous uncertain parameters within the hydraulic system. To address this issue, a ball screw is utilized to replace the original valve-controlled cylinder for swash plate control, enhancing accuracy and responsiveness. In addition, an in-depth analysis of the Ball Screw Variable Mechanism (BSVM) is conducted, leading to the development of a coupled mechanical–hydraulic dynamic model. Based on this model, a controller is designed to improve system performance. Finally, the effectiveness and high performance of the proposed new structure and control strategy were validated through comparative experiments and simulations. The experimental results confirm the advantages of the proposed design, demonstrating satisfactory improvements in control precision.

## 1. Introduction

In the context of global climate change and environmental challenges, issues pertaining to energy consumption and environmental pollution are becoming increasingly urgent. Hydraulic systems, renowned for their high power density, rapid response times, safety reliability, and adaptability, are extensively utilized as a means of power transmission in the industrial and machinery sectors [1]. Consequently, research into energy-efficient hydraulic technologies is critically important for enhancing energy efficiency, reducing operational costs, and mitigating environmental impacts [2].

Currently, based on the characteristics of hydraulic circuits, hydraulic systems can be divided into valve-controlled systems and (DC) systems, with valve-controlled systems being the most widely applied [3]. Valve-controlled systems are preferred for their low implementation cost, fast dynamic response, and high control precision. However, their significant energy consumption, caused by throttling losses, remains a major limitation. To eliminate throttling losses, DC systems have been developed, which adjust the flow rate to hydraulic actuators by modulating either the drive speed or pump displacement [4]. Nonetheless, DC systems still face challenges with dynamic response speed, system stability, and control precision. These limitations partially offset their energy efficiency advantages, thereby impacting the broader application of DC systems in various fields [5].

As the core power component of the DC system, the axial piston variable pump achieves flow regulation by adjusting the angle of the swashplate, enabling the precise control of actuator speed and position. Consequently, the axial piston variable pump plays a critical role in determining the overall flow control performance of the system [6]. This pump regulates system flow output through the swash plate, making the precise control of the swash plate essential to system performance. However, due to the high nonlinearity of the hydraulic system, the presence of external disturbances, and the complexities of modeling uncertainties [7,8], controlling the core component of the tilting plate—namely, the valve-controlled cylinder—becomes increasingly challenging. These factors lead to low control accuracy and limit its application scope. Joerg Grabbel et al. [9] designed a proportional control with second-order feedforward compensation and used the Ackermann parameter space method to design a robust controller, aiming to enhance swash plate control performance by accommodating servo valve parameter uncertainties. He Wei et al. [10] proposed an improved swash plate control system based on an asymmetric valve-controlled asymmetric piston approach, demonstrating its effectiveness in mitigating the negative impact of swash plate torque on the system’s dynamic performance. Helian Bobo et al. [11] developed an improved reduced-order dynamic modeling method and developed an adaptive robust backstepping controller to enable the accurate tracking of the swash plate’s target angle. Yang Song et al. [12] designed a control strategy based on the swash plate, proposing a new algorithm based on PI combined with feed-forward compensation to improve the dynamic performance.

The above studies primarily focus on enhancing the dynamic performance of variable pumps through advanced control algorithms. However, some researchers have fundamentally modified the traditional structure of variable pumps to improve their performance. For example, Reference [13] describes a variable floating cup pump that adjusts flow by altering the rotor speed or displacement, enabling precise flow control. This approach offers an expanded perspective for achieving accurate displacement control.

Ball screws, known for their rapid response, high precision, and compact size [14], are increasingly applied in fields such as robotics, flexible joints, aerospace, space manipulators, and CNC machine tools [15]. Compared to valve-controlled variable cylinders, ball screws eliminate certain hydraulic components, simplifying the hydraulic circuit and avoiding the complexity associated with extensive piping and additional hydraulic elements. Furthermore, the absence of fluid in the transmission path enhances stiffness and conversion efficiency under similar conditions. In contrast to traditional valve-controlled cylinders, ball screws also facilitate the implementation of advanced control strategies, enabling higher precision in control [16].

Thus, a novel variable displacement mechanism could be designed using a ball screw to achieve precise displacement control. Given the critical role of ball screw systems in achieving accurate displacement control [17], an in-depth analysis of their dynamic characteristics and the design of appropriate controllers are essential for ensuring system performance.

Traditional dynamic modeling methods for ball screw drives use lumped parameter models, combining the longitudinal and torsional flexibility of the screw with the equivalent stiffness of the ball nut, support bearings, and couplings [18,19,20]. However, lumped modeling approaches struggle to accurately capture the high-frequency dynamic characteristics of ball screw drive systems [21]. Okwudire et al. [22] analyzed the dynamic characteristics of ball screw feed systems using a hybrid model that considers the flexibility of the screw shaft. The multibody dynamic approach [23] is recognized as an effective compromise between simulation time and accuracy, enabling a detailed analysis of the characteristics of ball screws. Furthermore, it highlights the critical importance of proper mounting and load distribution in ensuring the rigidity, performance, and expected service life of the ball screw system. Reference [24] derived a parametric expression for the equivalent stiffness of the ball screw via finite element analysis. Based on the equivalent stiffness of the ball screw shaft, a ball screw drive dynamic model was established, incorporating the effects of bearing stiffness, nut mass, and axial preload.

In terms of precision control strategies for ball screw drives, Erkokmaz [25] and Dan [26] proposed a pole placement technique that enables active vibration damping, high-bandwidth disturbance rejection, and precise positioning. Hanifzadegan et al. [27,28] developed a linear parameter-varying (LPV) model and controller based on a linear time-varying parameter model of the ball screw feed system, utilizing gain scheduling to address time-varying characteristics, achieving favorable control performance. Dong L et al. [29] designed an adaptive sliding mode controller based on a flexible body model of the ball screw, estimating time-varying uncertainties within the system model and compensating for unknown external boundary disturbances. Munoa et al. [30] introduced a control loop using acceleration feedback, effectively achieving active vibration suppression.

In this paper, a novel variable mechanism based on a ball screw structure to achieve precise displacement control is proposed. Initially, the original valve-controlled cylinder mechanism is analyzed, identifying limitations in control accuracy and responsiveness. To address these issues, a ball screw is introduced to control the swash plate in place of the valve-controlled cylinder, enhancing both system accuracy and dynamic response. Through detailed analysis, the dynamic, hydraulic, and stiffness models of the system are established. Based on these models, a controller is designed that incorporates sliding mode control along with velocity and acceleration feedforward compensation to optimize performance. Simulation and experimental comparisons demonstrate that the new structure, combined with the proposed controller, offers superior performance over conventional methods. This study primarily focuses on enhancing the displacement output accuracy of axial piston pumps by employing ball screw mechanisms and model-based control strategies.

As shown in Table 1, the definitions of the symbols and descriptions are as follows.

## 2. Structure and Analysis of the Ball Screw Variable Mechanism (BSVM) System

### 2.1. Analysis of the Valve-Controlled Cylinder Variable Mechanism (VCCVM)

An in-depth analysis of the traditional VCCVM is conducted by introducing the Trajectory Sensitivity Analysis Function (TSAF) method [31] to quantitatively evaluate the impact of system parameter variations on the displacement of the variable piston cylinder. The displacement changes in the variable piston cylinder directly reflect changes in the swash plate angle, which is crucial for achieving precise displacement adjustment within hydraulic systems. The sensitivity analysis of the variable mechanism reveals the dynamic relationship between system parameters and output response, providing a theoretical foundation and guidance for the design and analysis of new variable mechanisms.

**Figure 1:**
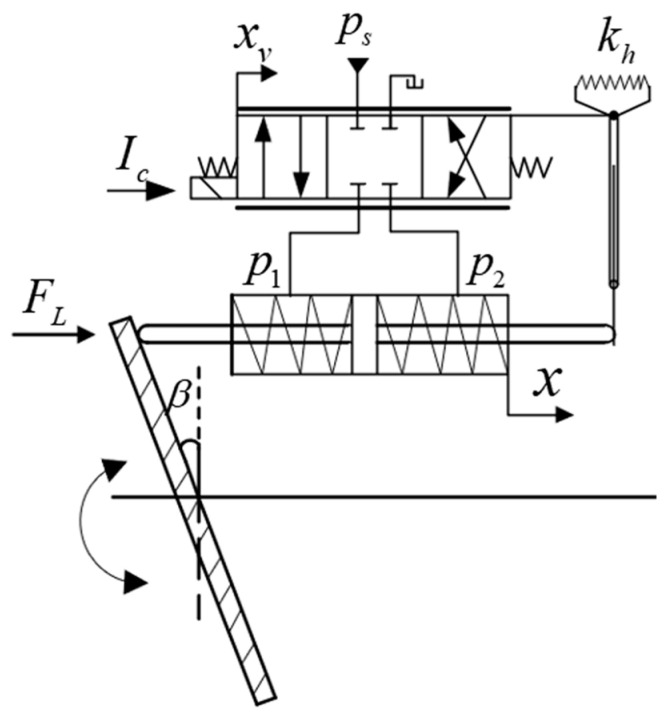
Schematic diagram of VCCVM system components.

The traditional variable mechanism, as shown in Figure 1, primarily consists of an electro-hydraulic proportional valve, a variable cylinder, a swash plate, and a displacement sensor. The control valve regulates the oil flow entering the variable cylinder, adjusts the direction of the oil flow to the cylinder, and controls the position of the variable cylinder.

If the return oil pressure *pr* is neglected and the spool displacement is assumed to be proportional to the input signal, the output flow rate equation for an electromagnetic proportional valve can be given as follows:

(1) $$Q_{L}=K_{t}u\sqrt{p_{s}-sign\left(u_{1}\right)p_{L}}$$

The flow continuity equation for the variable cylinder can be expressed as follows:

(2) $$Q_{L}=A\frac{dx}{dt}+C_{tc}p_{L}+\frac{V_{t}}{4\beta_{e}}\cdot \frac{dp_{L}}{dt}$$

The dynamic force balance equation for the variable cylinder is expressed as follows:

(3) $$Ap_{L}=m\ddot{x}+c\dot{x}+kx+F_{L}$$

Neglecting the fluid mass in the hydraulic pipelines, frictional losses, and fluid dynamics within the system, and based on Equations (1)–(3), the state variables are defined as $x=\left[x_{1}\;\;x_{2}\;\;x_{3}\right]=\left[x\;\;\;\dot{x}\;\;\frac{Ap_{L}}{m}\right]$, and the state equations for the variable control mechanism system can be obtained as follows:

(4) $$\left[\begin{array}{c} {\dot{x}}_{1}\\ {\dot{x}}_{2}\\ {\dot{x}}_{3} \end{array}\right]=\left[\begin{array}{c} x_{2}\\ x_{3}-\eta_{2}x_{1}-\eta_{1}x_{2}-\eta_{3}\\ \eta_{4}u_{1}g\left(u_{1},x_{3}\right)-\eta_{5}x_{2}-\eta_{6}x_{3} \end{array}\right]$$

The output equation is given as follows:

(5) $$y=\left[1\;\;0\;\;0\right]x$$

where $\;\eta_{1}=c/m$; $\eta_{2}=k/m$; $\eta_{3}=F_{L}/m$; $\eta_{4}=\left(4A\beta_{e}K_{t}\right)/\left(mV_{t}\right)$; $\eta_{5}=\left(4A^{2}\beta_{e}\right)/\left(mV_{t}\right)$; $\eta_{6}=\left(4c\beta_{e}C_{t}\right)4c\beta_{e}C_{t}/V_{t}$; and $g\left(u_{1},x_{3}\right)=\sqrt{p_{s}-sign\left(u_{1}\right)\frac{m}{A}x_{3}}$.

In hydraulic systems, the state–space equations for the variable mechanism can be represented in the general form as follows:

(6) $$\dot{x}=G\left(x,a,u,t\right)$$

where *x* represents the system state variable; a is a p-dimensional system parameter independent of *e*, where *e* is an r-dimensional input vector; and *t* denotes time. $x={\left[x_{1}\;\;x_{2}\;{\;x}_{3}\right]}^{T}$ represents the three state vectors of the variable mechanism system. Following the analysis of the variable structure system in Section 2.1, we selected six primary parameters for detailed investigation, $a={\left[a_{1}\;\;a_{2}\;{\;a}_{3}\;{\;a}_{4}\;\;a_{5}\;\;a_{6}\right]}^{T}$; $a_{1}=m$; $a_{2}=c$; $a_{3}=k$; $a_{4}=A$; $a_{5}=p_{s}$; and $a_{6}=p_{n}$.

The first-order trajectory sensitivity function is defined as follows:

(7) $$\lambda_{n}^{i}={\left(\frac{\partial x}{\partial a_{i}}\right)}_{n}$$

which represents the effect of changes in the parameters on the state variable.

Taking the partial derivative of both sides of the state–space equation G with respect to the parameter *a*, and considering that the input vector *e* and the parameter *a* are independent, the partial derivative of the input vector *e* with respect to the system parameter *a* is zero. Thus, we obtain the following: ${\left(\frac{\partial \dot{x}}{\partial a_{i}}\right)}_{n}=\frac{\partial G}{\partial a_{i}}+{\left(\frac{\partial G}{\partial x}\right)}_{n}\cdot {\left(\frac{\partial x}{\partial a_{i}}\right)}_{n}$. Substituting this into Equation (7), we obtain the following:

(8) $${\dot{\lambda }}_{n}^{i}={\left(\frac{\partial G}{\partial x}\right)}_{n}\cdot \lambda_{n}^{i}+{\left(\frac{\partial G}{\partial a_{i}}\right)}_{n}$$

Due to the small variation $\Delta a_{i}$ in the system parameter $a_{i}$, the resulting change Δx in the state variable *x* can be approximated using the Taylor expansion as follows:

(9) $$x{\left(t,a+\Delta a\right)}_{n}=x{\left(t,a\right)}_{n}+{\left(\frac{\partial x(t,a)}{\partial a}\right)}_{n}\cdot \Delta a+o(a)$$

Here, ${\left(\frac{\partial x(t,a)}{\partial a}\right)}_{n}$ represents the first-order trajectory sensitivity function $\;\lambda_{n}^{i}$, and we obtain the following:

(10) $$\Delta x=\lambda_{n}^{i}\cdot \Delta a+ο\left(a\right)$$

Equation (10) is the first-order expression of the change in state variable Δx caused by the change in parameter vector Δa. According to the equation, as long as the first-order trajectory sensitivity function $\lambda_{n}^{i}$ of each parameter is calculated and multiplied with the change in parameter Δa, the change in state variable Δa, caused by the change in each parameter, can be calculated.

By taking the partial derivatives of Equation (6) with respect to the system state variable *x* and system parameter *a*, the trajectory sensitivity functions $\lambda_{n}^{i}$ can be computed. Once the trajectory sensitivities for each parameter in the variable control system are obtained, a quantitative sensitivity measure *S* is introduced to analyze the impact of parameter variations on piston displacement. This approach enables a systematic assessment of how changes in individual parameters affect the displacement of the piston cylinder.

The measurement index *S* is now defined as follows:

(11) $$S=mean\left(\bar{a_{1}}+\bar{a_{2}}\right)$$

where

(12) $$\bar{a_{1}}=\max\left(x_{1}\right)-\max\left(x_{1}-\lambda_{1}^{i}\cdot \Delta a_{i}\right)\;\bar{a_{2}}=\min\left(x_{1}-\lambda_{1}^{i}\cdot \Delta a_{i}\right)-\min\left(x_{1}\right)$$

**Figure 2:**
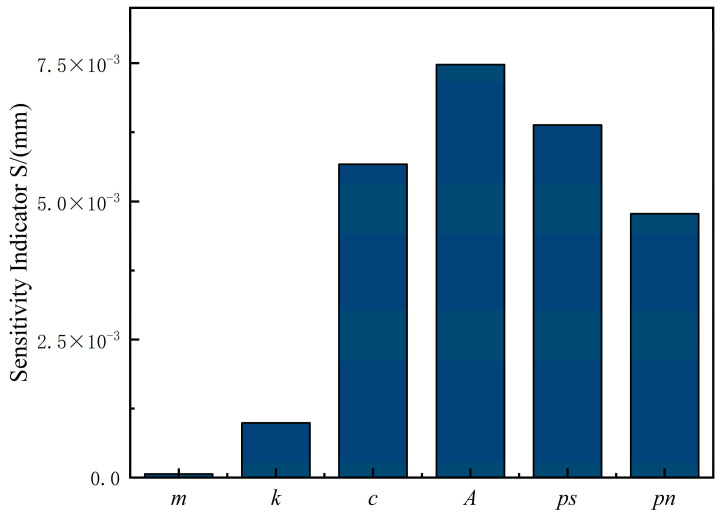
Sensitivity measurement index *S* value when each parameter of the system changes by 10%.

Based on the model setup and simulation described above, a sinusoidal signal was selected as the input signal for the sensitivity analysis, with the displacement of the variable piston cylinder serving as the basis for the analysis. The resulting displacement curve of the variable piston cylinder, induced by a 10% variation in the system parameter within the variable control system, is presented in Figure 2.

From Figure 2, it can be concluded that the effective area of the piston cylinder A, the control oil source pressure $p_{s}$, the viscous friction coefficient c, the external load $p_{n}$, and the system spring stiffness k are the key parameters influencing the piston cylinder’s displacement. In contrast, the impact of mass m has little effect on displacement. This analysis aims to optimize the performance of the valve-controlled cylinder variable mechanism by adjusting the internal system parameters. However, it should be noted that $p_{s}$ and $p_{n}$ are externally determined by the load and the system configuration, while A and c are often constrained by physical structures, material properties, and oil viscosity. These parameters are inherently difficult to modify. Due to the intrinsic limitations of our existing structure, further improvements are exceedingly difficult to achieve. Therefore, we propose a novel structure to overcome these challenges and achieve superior performance.

### 2.2. Structure and Operating Principle of the Ball Screw Variable Mechanism (BSVM) System

**Figure 3:**
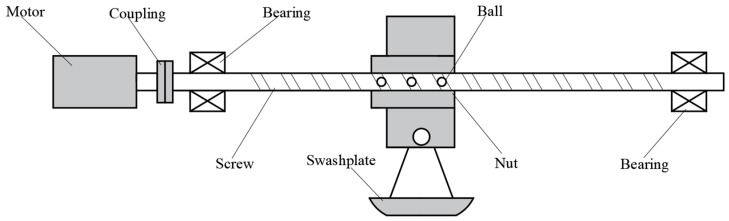
Schematic diagram of BSVM components.

To enable precise control of pump displacement, a novel variable mechanism system is proposed. The schematic of this new mechanism, shown in Figure 3, consists of a permanent magnet synchronous motor (PMSM), coupling, ball screw, nut assembly, support bearings, fixed bearings, and a swash plate. The PMSM provides precise rotational power and enables accurate control of the ball screw by precisely regulating speed and torque. The system employs an elastic coupling to transmit torque from the motor to the ball screw while compensating for minor misalignments caused by installation errors or operational deviations and absorbing vibrations. The bearing configuration adopts a fixed-support mounting scheme, which guides and supports the ball screw. Angular contact ball bearings are utilized, as they are well-suited for high-speed and precision applications, effectively reducing friction and wear. The ball screw converts the motor’s rotational motion into linear motion of the nut assembly, which in turn adjusts the tilt angle of the swash plate, ultimately controlling the displacement of the axial piston pump. In the proposed design, the nut and swash plate are connected via a slider. The two sides of the slider are constrained within grooves specifically machined on the nut sleeve, while the hole on the slider is fitted onto the swash plate shaft. When the nut moves laterally, the force is transmitted to the shaft of the swash plate. This shaft is offset by a distance *L* from the swing axis of the swash plate, generating a torque that causes the swash plate to oscillate. Based on the displacement *x* of the nut and the offset *L* of the swash plate’s hinge relative to the swing axis, the relationship between the swash plate swing angle and these two parameters can be derived as follows:

(13) $$\beta =arcsin\frac{x}{L}$$

### 2.3. Mechanical Dynamics Model

**Figure 4:**
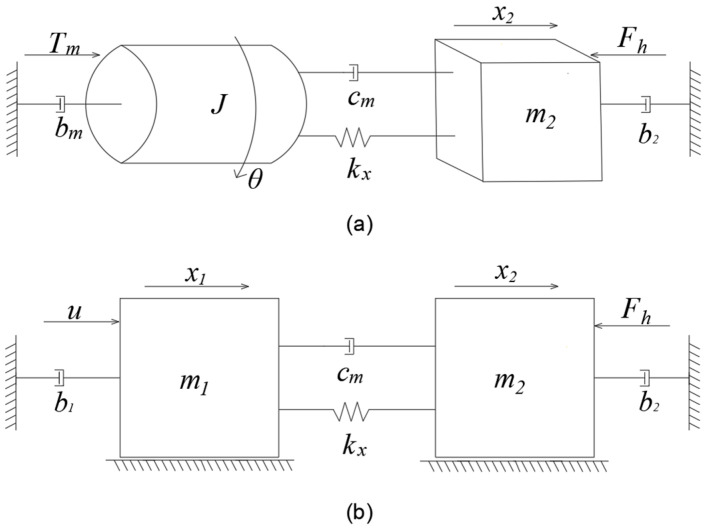
Mechanical system dynamics model: (**a**) Rotary-to-linear motion model with torque driving the system; (**b**) Equivalent translational model.

Based on the ball screw system, the model is simplified as a “spring-mass-damper” system, reducing the structural complexity to a two-degree-of-freedom model [32]. In Figure 4, Model (a) is intuitively decomposed into two fundamental motion components as follows: the rotational motion around the motor axis and the axial translational motion along the linear guide. To facilitate the controller design in the following sections, the screw transmission ratio $r_{g}=h/2\pi$ is used to convert the physical quantities of rotational motion into their equivalent linear motion counterparts, resulting in a comprehensive dynamic model, as shown in Model (b) [33,34].

Based on Figure 4, the mechanical motion equation can be written as:

(14) $$u=m_{1}\ddot{x_{1}}+b_{1}\dot{x_{1}}+\left(\dot{x_{1}}-\dot{x_{2}}\right)c_{m}+\left(x_{1}-x_{2}\right)k_{x}\;F_{h}=m_{2}\ddot{x_{2}}+b_{2}\dot{x_{2}}+\left(\dot{x_{2}}-\dot{x_{1}}\right)c_{m}+\left(x_{2}-x_{1}\right)k_{x}$$

where $m_{1}$ represents the equivalent mass of the rotational components, and $m_{2}$ denotes the equivalent mass of the linear components. The parameter $b_{1}$ is the damping coefficient of the rotational components, $k_{x}$ is the axial stiffness of the ball screw, $c_{m}$ represents the viscous coefficient within the ball screw and nut assembly, and $b_{2}$ is the damping coefficient of the moving components. The term $F_{h}$ is the force exerted by the swash plate on the variable mechanism, while u represents the motor input torque. Equation (14) can be expressed in the Laplace domain as follows:

(15) $$G_{t}\left(s\right)=\frac{x_{2}\left(s\right)}{u\left(s\right)}=\frac{c_{m}s+k_{x}}{\eta }\;G_{n}\left(s\right)=\frac{x_{1}\left(s\right)}{u\left(s\right)}=\frac{m_{2}s^{2}+\left(b_{2}+c_{m}\right)s+k_{x}}{\eta }$$

where

(16) $$\eta =m_{1}m_{2}s^{4}+\left[m_{2}\left(b_{1}+c_{m}\right)+m_{1}\left(b_{2}+c_{m}\right)s^{3}\right]\;+\left[b_{1}b_{2}+\left(b_{1}+b_{2}\right)c_{m}+\left(m_{1}+m_{2}\right)k_{x}\right]s^{2}+k_{x}\left(b_{1}+b_{2}\right)s$$

$G_{t}(s)$ and $G_{n}(s)$ are the transfer functions representing the relationships between the motor torque and motor position and between the motor torque and displacement of the variable mechanism, respectively. Equation (14) describes the overall transfer dynamics of the ball screw system in the form of transfer functions.

### 2.4. Analysis of Forces on the Swash Plate

For the variable mechanism, the total torque acting on the swash plate is regarded as an external disturbance. This disturbance not only impacts the control accuracy of the mechanism but can also cause vibrations and noise during torque fluctuations. Consequently, the dynamic characteristics of the swash plate torque have a significant impact on the response speed and control precision of the variable mechanism. To address this, we analyze the torque on the swash plate and establish a coupled mechanical–hydraulic dynamic model. The total torque $M_{x}\;$ acting on the swash plate primarily consists of two main components as follows: the unbalanced hydraulic torque $M_{p}$, generated by the hydraulic pressure at the base of the plungers acting on the swash plate through the plunger shoes; and the inertial torque ${\;M}_{g}$, caused by the reciprocating inertial forces of the plunger and shoe assembly.

The force exerted on the variable mechanism by the swash plate torque is expressed as follows:

(17) $$F_{h}=\frac{M_{x}\cos\beta }{L}$$

**Figure 5:**
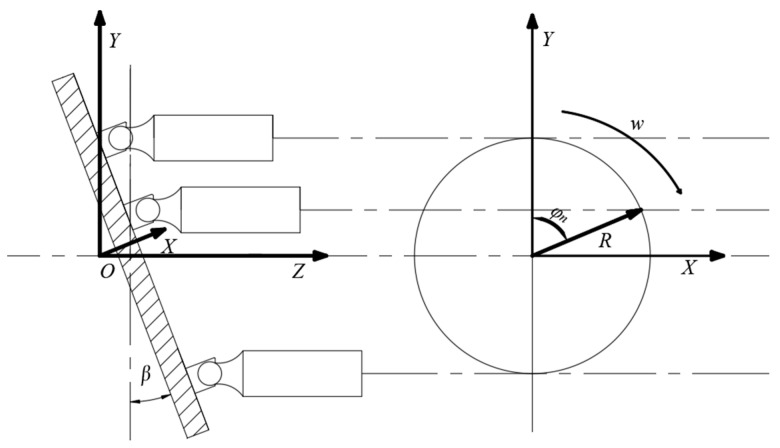
Schematic diagram of the force exerted by the plunger on the swash plate.

To facilitate the analysis of the forces and resulting torques on the swash plate, it is necessary to establish a spatial Cartesian coordinate system, as illustrated in Figure 5. The XYZ coordinate system is established with the intersection point of the swash plate and the main shaft as the origin. The *Z*-axis is aligned with the rotational axis of the main shaft and points positively toward the valve plate. The *Y*-axis is oriented vertically, while the rotational axis of the main shaft defines the central axis of the coordinate system.

The expression for the hydraulic torque $M_{p}$ on the swash plate due to hydraulic pressure is given by the following:

(18) $$M_{p}=\frac{\pi d^{2}R}{4\cos^{2}\;\beta }\sum_{n=1}^{N}p_{n}\cos\varphi_{n}$$

Due to variations in the cylinder angle, the number of plungers within the high-pressure discharge region will change accordingly, expressed as follows:

(19) $$M_{p}=\left\{\begin{array}{l} \frac{\pi d^{2}R}{4{cos}^{2}\beta }\sum_{n=1}^{N}p_{n}cos\varphi_{n},\;0\leq \varphi_{n}\leq \frac{\alpha }{2}\\ \frac{\pi d^{2}R}{4{cos}^{2}\beta }\sum_{n=1}^{N-1}p_{n}cos\varphi_{n},\;\;\frac{\alpha }{2}\leq \varphi_{n}\leq \alpha \end{array}\right.$$

The hydraulic pressure $p_{n}$ varies with the cylinder angle and can be represented as follows:

(20) $$p_{n}=\left\{\begin{array}{l} p_{h}\;\;0\leq \varphi_{n}\leq \pi \\ p_{l}\;\;\pi \leq \varphi_{n}\leq 2\pi \end{array}\right.$$

where $p_{h}$ represents the hydraulic pressure on the plunger in the high-pressure region, and $p_{l}$ represents the hydraulic pressure on the plunger in the low-pressure region.

The torque $M_{g}$ is expressed as follows:

(21) $$M_{g}=\frac{m_{z}\omega^{2}R^{2}\tan\beta }{\cos^{2}\;\beta }\sum_{n=1}^{N}\cos^{2}\;\varphi_{n}$$

### 2.5. Stiffness Analysis of the Variable Mechanism

Based on the analysis of traditional structures in Section 2.1, the proposed new structural model significantly simplifies the parameters by eliminating many variables present in conventional designs. Furthermore, as discussed in Section 2.4, vibrations are induced by torque fluctuations in the swashplate. This is one of the reasons we utilize a ball screw, as its high stiffness helps reduce the vibration amplitude. As a result, we conducted an analysis of the stiffness of the ball screw. Therefore, a detailed study of the stiffness characteristics of the new variable mechanism is warranted. In this study, a stiffness model of the variable mechanism is developed, considering the stiffness characteristics of the screw, nut, and bearings. The axial stiffness of the variable mechanism is expressed as follows:

(22) $$\frac{1}{k_{x}}=\frac{1}{k_{l}}+\frac{1}{k_{n}}+\frac{1}{k_{b}}$$

where $k_{l}\;$ represents the axial stiffness of the ball screw, $k_{n}\;$ denotes the axial stiffness of the nut assembly, and $k_{b}$ is the axial stiffness of the bearing assembly.

With the ball screw installed in a configuration where one end is fixed and the other end is supported, the axial stiffness of the ball screw can be derived as follows:

(23) $$k_{l}=\frac{\pi Ed^{2}}{4}\left(\frac{L_{b}}{x_{b}}\right)$$

**Figure 6:**
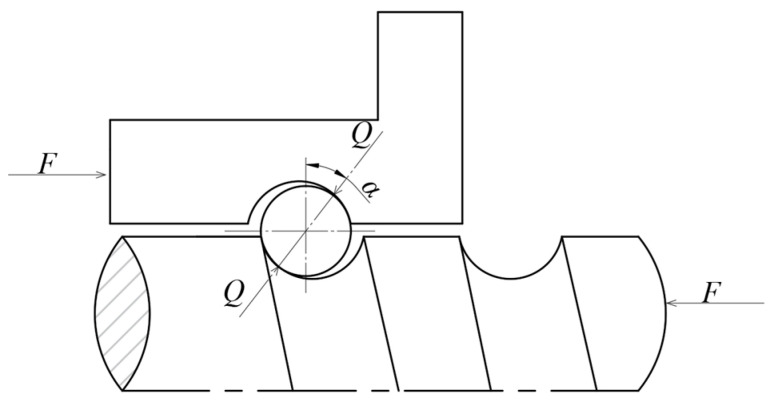
Force deformation diagram of a single ball.

The axial stiffness of the nut assembly is determined by the ratio of the applied axial load to the corresponding axial deformation. This axial deformation primarily arises from the elastic contact deformation between the balls and the raceway surface, which can be theoretically calculated using Hertzian contact theory. Assuming that the contact angles between the balls and the screw, as well as between the balls and the nut, are constant and equal, the force diagram of the balls can be represented as shown in Figure 6. The total normal load on all balls is denoted as $Q=zQ_{1}$, where *z* represents the number of balls, and $Q_{1}$ is the normal load on a single ball. Consequently, the relationship between the cumulative normal load of the load-bearing balls and the axial thrust *F* applied to the nut is defined as follows:

(24) $$Q=\frac{F}{z\sin\alpha \cos\gamma }$$

Neglecting the effects of uneven load distribution, the elastic deformation of a single ball under the normal load Q is illustrated in Figure 5. The normal elastic displacement resulting from the elastic contact deformation of the ball can be determined based on Hertzian contact theory, as follows:

(25) $$\delta_{n}=\delta_{1}+\delta_{2}={\left(\frac{3Q}{2E^{\prime }}\right)}^{\frac{2}{3}}\left[\delta_{1}^{*}{\left(\sum \rho_{1}\right)}^{\frac{1}{3}}+\delta_{2}^{*}{\left(\sum \rho_{2}\right)}^{\frac{1}{3}}\right]$$

where $\delta_{1}$ represents the elastic deformation at the contact interface between the ball and the screw, while $\delta_{2}$ denotes the elastic deformation at the contact interface between the ball and the nut. *E*′ refers to the effective modulus of elasticity. The dimensionless parameters $\delta_{1}^{*}$ and $\delta_{2}^{*}$ are related to the contact ellipse parameters for the nut and the screw, respectively. It is worth noting that the calculation of $\rho_{1}$ and $\rho_{2}$ depends not only on the contact angle but also on the helix angle [35], which differentiates it from the subsequent calculation of bearing stiffness.

The comprehensive curvature at the contact points between the ball and the screw, as well as the ball and the nut, are represented as $\rho_{1}$ and $\rho_{2}$, respectively, as follows:

(26) $$\left\{\begin{array}{c} \sum \rho_{1}=\frac{2}{d_{n}}+\frac{2}{d_{n}}-\frac{1}{d_{n}f_{n1}}+\frac{2cos\alpha sin\gamma }{d_{n1}-d_{n}cos\alpha }\\ \sum \rho_{2}=\frac{2}{d_{n}}+\frac{2}{d_{n}}-\frac{1}{d_{n}f_{n2}}-\frac{2cos\alpha sin\gamma }{d_{n1}+d_{n}cos\alpha } \end{array}\right.$$

where $d_{n}$ and $d_{n1}$ represent the diameters of the ball and the screw, respectively. $f_{n1}$ denotes the ratio of the screw curvature radius to the ball radius, while $f_{n2}$ represents the ratio of the nut curvature radius to the ball radius.

The normal elastic displacement between the nut and the screw along the axis results in the axial elastic displacement of the nut relative to the screw. The relationship between these displacements is given by the following:

(27) $$\delta_{a}=\delta_{n}\frac{\cos\gamma }{\sin\alpha }$$

By substituting Equations (24) and (25) into Equation (27), the axial elastic displacement of the single-nut ball screw assembly can be obtained as follows:

(28) $$\delta_{n}={\left(\frac{3F}{2{zE}^{\prime }}\right)}^{\frac{2}{3}}\left[\delta_{1}^{*}{\left(\sum \rho_{1}\right)}^{\frac{1}{3}}+\delta_{2}^{*}{\left(\sum \rho_{2}\right)}^{\frac{1}{3}}\right]{\left(\sin\alpha \right)}^{\frac{5}{3}}{\left(\cos\gamma \right)}^{\frac{1}{3}}$$

By differentiating both sides of Equation (28) with respect to the axial displacement, the axial stiffness of the nut assembly can be obtained as follows [36]:

(29) $$k_{n}=\frac{dF}{d\delta_{a}}=\frac{3F^{\frac{1}{3}}}{{\left(\frac{3}{2zE^{\prime }}\right)}^{\frac{2}{3}}\left[\delta_{1}^{*}{\left(\sum \rho_{1}\right)}^{\frac{1}{3}}+\delta_{2}^{*}{\left(\sum \rho_{2}\right)}^{\frac{1}{3}}\right]{\left(\sin\alpha \right)}^{\frac{5}{3}}{\left(\cos\gamma \right)}^{\frac{1}{3}}}$$

Considering the structural and load similarities between bearings and ball screw assemblies, Hertzian contact theory is applicable for calculating the axial stiffness of the support system. Furthermore, the method used to calculate the axial contact stiffness of the ball screw assembly can also be applied to the axial stiffness analysis of the support system. The axial deformation of a single ball within the bearing can be calculated using Equation (24). The axial contact deformations between the balls and the inner and outer raceways, denoted as $\delta_{3}$ and $\delta_{4}$, can be obtained using Equation (25). The principal curvatures of the bearing, $\rho_{3}$ and $\rho_{4}$, can be calculated using Equation (25), but the helix angle of the screw must be neglected. The axial contact stiffness of the bearing is determined using Equation (29). The specific calculation formulas are as follows:

(30) $$\left\{\begin{array}{c} Q_{b}=\frac{F_{b}}{z_{1}sin\lambda }\\ \delta ={\left(\frac{3Q_{b}}{2{E_{b}}^{\prime }}\right)}^{\frac{2}{3}}\left[\delta_{3}^{*}{\left(\sum \rho_{3}\right)}^{\frac{1}{3}}+\delta_{4}^{*}{\left(\sum \rho_{4}\right)}^{\frac{1}{3}}\right]\\ k_{b}=\frac{3F_{b}^{\frac{1}{3}}}{{\left(\frac{3}{2z_{1}{E_{b}}^{\prime }}\right)}^{\frac{2}{3}}\left[\delta_{3}^{*}{\left(\sum \rho_{3}\right)}^{\frac{1}{3}}+\delta_{4}^{*}{\left(\sum \rho_{4}\right)}^{\frac{1}{3}}\right]{\left(sin\lambda \right)}^{\frac{1}{3}}} \end{array}\right.$$

where $Q_{b}$ denotes the normal load on the bearing assembly, and $F_{b}$ represents the axial preload applied to the bearing assembly. The variable $z_{1}$ indicates the number of load-bearing balls, and λ is the contact angle between the balls and the raceway. The term ${E_{b}}^{\prime }$ represents the effective elastic modulus, while $\delta_{3}^{*}$ and $\delta_{4}^{*}$ are dimensionless parameters related to the contact ellipse parameters for the inner and outer bearing rings, respectively. The terms $\sum \rho_{3}$ and $\sum \rho_{4}$ denote the sum of the principal curvatures for the inner and outer raceways of the bearing, respectively.

## 3. Controller Design

**Figure 7:**
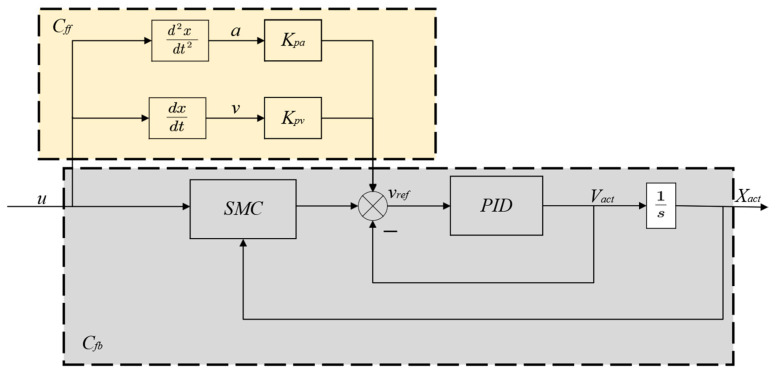
System control block diagram.

In this section, we propose a Velocity Feedforward plus Sliding Mode Control (VFSMC) strategy for the BSVM system. The controller framework, as shown in Figure 7, is composed of the following two main components: A control feedback design ($C_{fd}$): A sliding mode controller was designed for the mechanical–hydraulic model of the ball screw to eliminate the swash plate disturbance discussed in Section 2.4, enabling precise control. A control feedforward design ($C_{ff}$): Considering the frequent directional changes required by the variable mechanism of the axial piston pump under actual operating conditions, we introduce a feedforward compensation scheme for both velocity and acceleration. This scheme combines the feedforward compensation signal and the sliding mode controller output to generate a differential input, which is then processed by the PID controller to produce the final control signal.

### 3.1. Design of Sliding Mode Control

As described in Section 2.3, since the system’s characteristic equation is a fourth-order equation, four state variables are selected to express the system in state–space form. The state variables are chosen as $x=\left[x_{1}\;\;\dot{x_{1}}\;\;x_{2}\;\;\dot{x_{2}}\right]$. The driving model can thus be expressed in state–space representation as $\dot{x}=Ax+Bu+CF_{h}$ [37].

Where

(31) $$A=\left[\begin{array}{cccc} 0 & -\frac{k_{x}}{m_{1}} & 0 & \frac{k_{x}}{m_{1}}\\ 0 & -\frac{\left(c_{m}+b_{1}\right)}{m_{1}} & \frac{k_{x}}{m_{2}} & \frac{c_{m}}{m_{1}}\\ 0 & \frac{k_{x}}{m_{2}} & 0 & -\frac{k_{x}}{m_{2}}\\ 0 & \frac{c_{m}}{m_{2}} & 0 & -\frac{\left(c_{m}+b_{2}\right)}{m_{2}} \end{array}\right]\;B=\left[\begin{array}{c} 0\\ \frac{1}{m_{1}}\\ 0\\ 0 \end{array}\right]\;C=\left[\begin{array}{c} 0\\ 0\\ 0\\ \frac{1}{m_{2}} \end{array}\right]$$

The objective is to constrain the system states onto a stable sliding surface, ensuring that they ultimately converge to zero. The sliding surface is defined as follows:

(32) $$s=\lambda_{1}e_{1}+\lambda_{2}e_{2}+\lambda_{3}e_{3}+e_{4}$$

where, $e_{1}$, $e_{2}$, $e_{3}\;$ and $e_{4}$ represent the displacement and velocity deviations of the mechanism and the motor, respectively. $\bar{x_{1}}$, $\bar{x_{2}}$, $\bar{x_{3}}$ and $\bar{x_{4}}$ reference displacements and velocities of the motor and the ball screw are represented separately, leading to the following error definition:

(33) $$e_{1}=\bar{x_{2}}-x_{2},e_{2}=\bar{x_{1}}-x_{1},\;e_{3}=\bar{{\dot{x}}_{2}}-\dot{x_{2}},e_{4}=\bar{{\dot{x}}_{1}}-\dot{x_{1}}$$

The derivative of the sliding surface is expressed as follows:

(34) $$\begin{array}{ll} \dot{s} & =\lambda_{1}{\dot{e}}_{1}+\lambda_{2}{\dot{e}}_{2}+\lambda_{3}{\dot{e}}_{3}+{\dot{e}}_{4}\\ & =\lambda_{1}\left({\bar{\dot{x}}}_{2}-{\dot{x}}_{2}\right)+\lambda_{2}\left({\bar{\dot{x}}}_{1}-{\dot{x}}_{1}\right)\\ & +\lambda_{3}\left({\bar{\ddot{x}}}_{2}-\frac{1}{m_{2}}\left(-b_{2}{\dot{x}}_{2}+k_{x}\left(x_{1}-x_{2}\right)+c_{m}\left({\dot{x}}_{1}-{\dot{x}}_{2}\right)-F_{h}\right)\right)\\ & +{\bar{\ddot{x}}}_{1}-\frac{1}{m_{1}}\left(-b_{1}{\dot{x}}_{1}-k_{x}\left(x_{1}-x_{2}\right)-c_{m}\left({\dot{x}}_{1}-{\dot{x}}_{2}\right)+u\right) \end{array}$$

If the exponential reaching law is applied, it can be expressed as follows:

(35) $$\dot{s}=-\varepsilon \mathrm{sgn}s-rs$$

By combining Equations (34) and (35), the control law *u* is obtained as follows:

(36) $$\begin{array}{ll} u & =m_{1}\left(\lambda_{1}\left(\bar{{\dot{x}}_{2}}-\dot{x_{2}}\right)+\lambda_{2}\left(\bar{{\dot{x}}_{1}}-\dot{x_{1}}\right)\right)\\ & +\lambda_{3}\left(\bar{{\ddot{x}}_{2}}-\frac{1}{m_{2}}\left(-b_{2}\dot{x_{2}}+k_{x}\left(x_{1}-x_{2}\right)+c_{m}\left(\dot{x_{1}}-\dot{x_{2}}\right)-F_{h}\right)\right)\\ & +\bar{{\ddot{x}}_{1}}+\frac{b_{1}}{m_{1}}\dot{x_{1}}+\frac{k_{x}}{m_{1}}\left(x_{1}-x_{2}\right)+\frac{c_{m}}{m_{1}}\left(\dot{x_{1}}-\dot{x_{2}}\right)+\varepsilon \mathrm{sgn}s+ks \end{array}$$

### 3.2. Velocity and Acceleration Feedforward Compensation

To simplify the subsequent analysis, the flexible model described in Section 2.3 is reduced to a rigid model by neglecting the elastic deformation of mechanical components, including the motor spindle, coupling, lead screw, and nut. Assuming an axial stiffness $k_{x}=\infty$, the differential equation relating the axial position of the worktable x(t) to the control voltage u(t) can be derived. After applying the Laplace transform, the axial position of the worktable is expressed as follows:

(37) $$\frac{x\left(s\right)}{u\left(s\right)}=\frac{1}{ms^{2}}$$

A typical Single Input Single Output (SISO) system is commonly characterized as a combination of a rigid model at low frequencies and multiple flexible models at high frequencies. The rigid model is denoted as $C_{r}$, while the flexible model is denoted as ${\;C}_{f}$. Thus, the system transfer function $G\left(s\right)$ can be expressed as follows:

(38) $$G\left(s\right)=C_{r}\left(S\right)+C_{f}\left(s\right)=\frac{1}{ms^{2}}+\sum_{i=1}^{n_{f}}\frac{k_{i}}{m\left(s^{2}+2\xi_{i}\omega_{i}s+w_{i}^{2}\right)}$$

where *m* represents the total mass of the system, ${\;n}_{f}$ denotes the number of flexible models, ${\;\omega }_{i}$ is the natural frequency, $\xi_{i}$ is the damping ratio, and $k_{i}$ represents the system gain.

**Figure 8:**
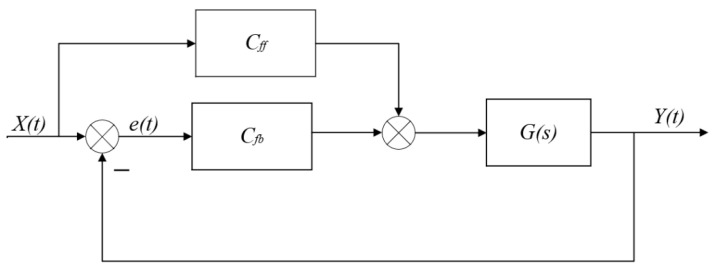
Two-degree-of-freedom control architecture.

By introducing the two-degree-of-freedom control architecture shown in Figure 8, the transfer function $G_{e}$ from the reference signal x(t) to the tracking error e(t) can be derived. Its specific mathematical form is as follows:

(39) $$G_{e}=\frac{1-G\left(s\right)C_{ff}}{1+C\left(s\right)C_{fb}}$$

The acceleration feedforward is set as the inverse of the rigid model of the controlled system, denoted as ${C_{r}}^{-1}$, which is represented by $ms^{2}$. For the rigid model of the controlled system, using acceleration feedforward yields $\;C_{r}{C_{r}}^{-1}=1$. Substituting this into Equation (39) results in $\;G_{e}=0$, indicating perfect tracking of the rigid model. However, actual motion systems are often more complex, necessitating the introduction of velocity feedforward to compensate for the dynamic damping term of the system.

## 4. Results of Experiments and Discussion

### 4.1. Experimental Setup

**Figure 9:**
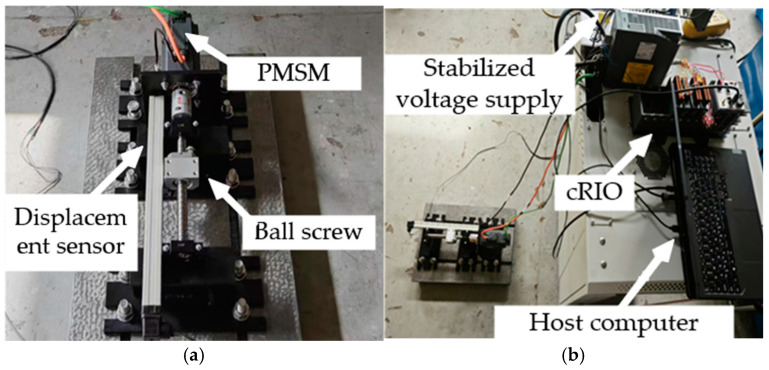
The experimental test rig: (**a**) ball screw test rig; (**b**) overall configuration of the rig.

**Figure 10:**
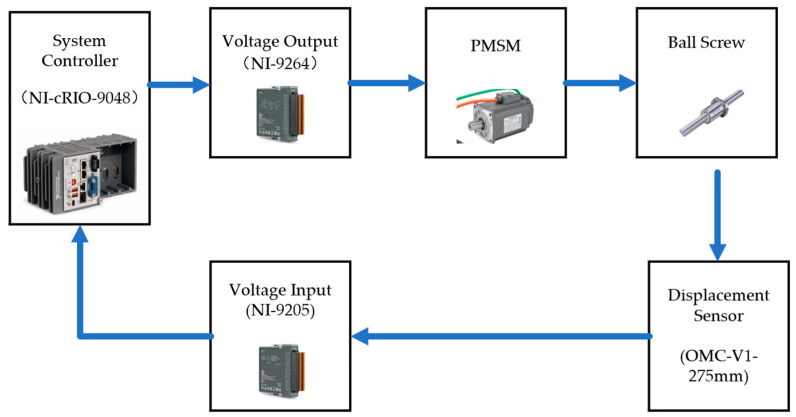
Schematic of system components.

The control system for the new variable mechanism is shown in Figure 9. To verify the effectiveness of the proposed device and control model, experiments were conducted using a ball screw transmission mechanism. A displacement sensor was connected to the NI-9025 board of the cRIO system, and the voltage output to the permanent magnet synchronous motor (PMSM) was managed through the NI-cRIO controller and the NI-9264 module. The control model execution and data storage are implemented using VeriStand 2024 Q3 software. Additionally, the schematic of the system connections is shown in Figure 10.

**Figure 11:**
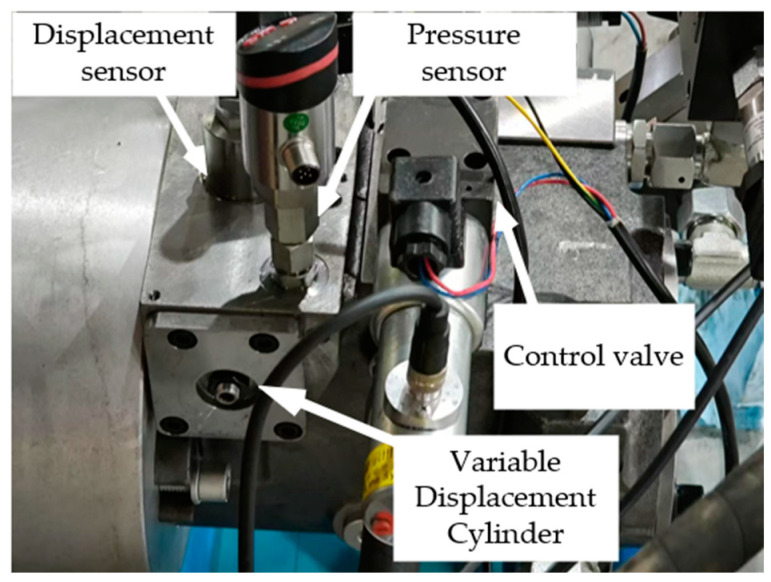
Valve-controlled variable mechanism test bench.

The test rig for the valve-controlled cylinder variable mechanism is shown in Figure 11, and its operating principle is as follows: The NI-cRIO control system outputs a ±10 V voltage signal to the amplifier, which drives the proportional valve. The proportional valve regulates the displacement of the spool to adjust the valve opening, thereby varying the flow rate through the valve-controlled system and driving the variable cylinder to achieve displacement control. A position sensor provides real-time feedback on the cylinder’s position to the NI-cRIO system, forming a closed-loop control system. Simultaneously, a pressure sensor is employed to monitor the system pressure in real time to ensure control accuracy and stability.

### 4.2. BSVM Simulation Verification

To validate the effectiveness of the proposed model, tests were conducted using the system simulation model built in the software, and the results were compared with actual measured data. The simulation model and the BSVM test bench were subjected to the same positive and negative switching step input signals in an open-loop test. This approach reflects the raw responses of the model and the test bench, providing a direct comparison of their fundamental behaviors. The simulation parameters are as follows: the lead is 5 mm, $m_{1}=13.2\;kg$,${\;m}_{2}=0.5\;kg$, ${\;k}_{x}=4.24\times 10^{7}\;N/m,$ ${\;b}_{1}=4.6572\;N/m\cdot s^{-1}$, ${\;b}_{2}=594.68\;N/m\cdot s^{-1}$ and ${\;c}_{m}=1727\;N/m\cdot s^{-1}.$

**Figure 12:**
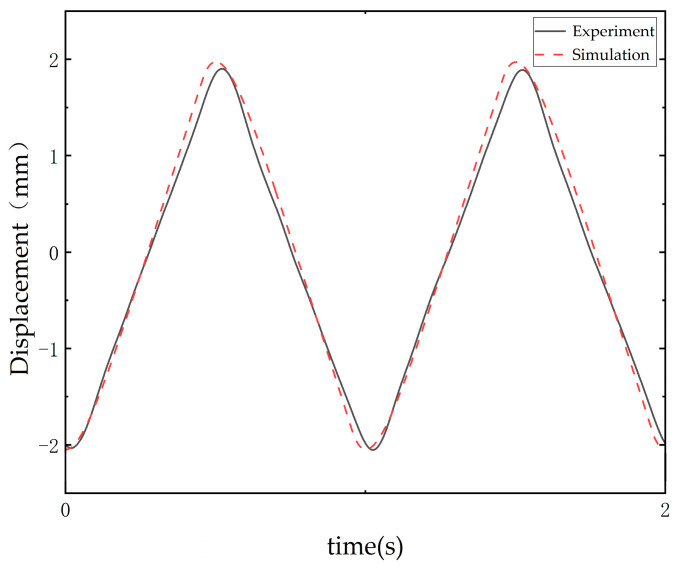
Simulation and experiment system response (without the actual swashplate connected).

The results are shown in Figure 12. From the figure, it can be observed that there are some errors at the peaks. This discrepancy may be attributed to potential mechanical nonlinearities within the system, such as backlash in the ball screw or elastic deformations, which could cause delays or offsets in the response, leading to the observed errors. At 10 s, the root mean square error between the simulation and experiment is 0.05 mm.

### 4.3. Comparative Experimental Study on the Performance of VCCVM and BSVM

In the experiment, the target displacement was set to ±8 mm, representing the maximum positive and negative displacement range for the variable mechanisms. The pump speed of the VCCVM test platform was set to 1250 rpm, with the control oil circuit pressure set to 4 MPa. The motor speed of the BSVM test platform was set to 1500 rpm. A PID controller was applied to both types of variable mechanisms, with parameters estimated through empirical tuning as follows: the VCCVM parameters were set to $k_{p}=2.5;k_{i}=0.3;k_{d}=0$; for the BSVM, the parameters were set to $k_{p}=0.2;k_{i}=0.1;k_{d}=0$.

**Figure 13:**
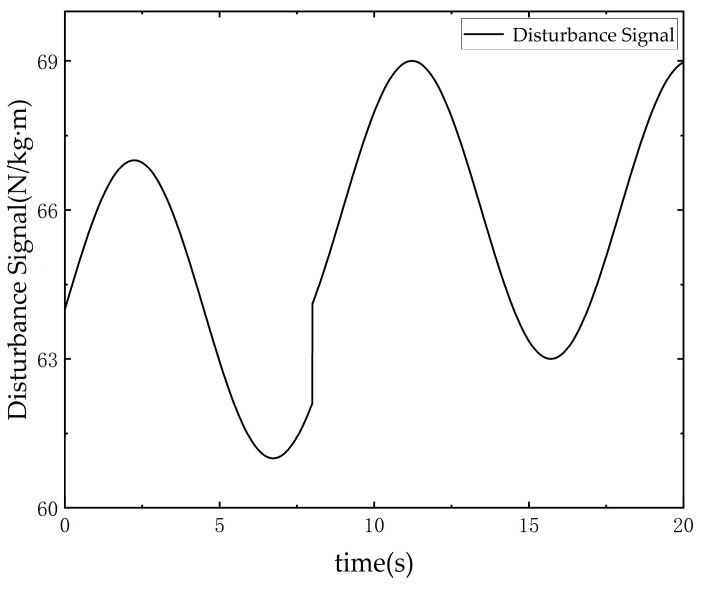
Equivalent disturbance signal used in the BSVM simulation.

Additionally, due to the differing environments of the two test benches, the VCCVM test bench operates under disturbance forces caused by the presence of oil, while the BSVM test bench does not account for external forces. To ensure equivalent testing conditions for both systems, the viscous resistance of the BSVM system in the presence of oil, as well as nonlinearities such as oil source fluctuations and temperature variations, were considered. The added equivalent interference force $F_{d}$ is shown in Figure 13. The equivalent disturbance force was input into the model to obtain the resulting displacement variation, and the corresponding disturbance displacement curve was directly incorporated into the experimental procedure. Considering the directional effects during motion, the disturbance signal was adjusted to $sgn\left(x_{2}\right)\cdot F_{d}$.

**Figure 14:**
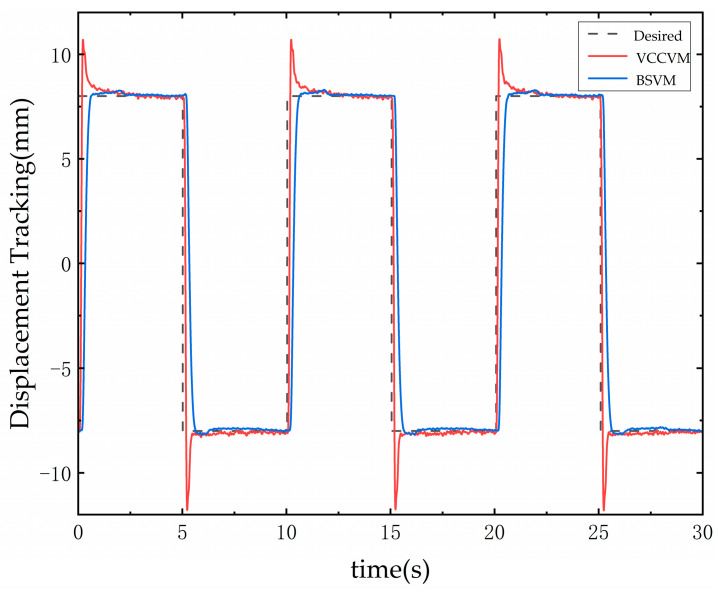
Step response tracking curve for positive and negative full displacement switching.

The tracking responses of both systems are shown in Figure 14. According to the test results, with a response error tolerance of ±0.1 mm, the BSVM demonstrated a 29% reduction in overshoot and a 0.6-s faster settling time compared to the VCCVM. When the displacements of both systems stabilized, the measured error for the BSVM system was ±0.06 mm, while the error for the VCCVM system was ±0.085 mm. Based on the obtained error and using Equation (13), along with the flow formula $Q_{t}=v_{t}\cdot n$, the flow error for the BSVM is calculated to be 0.57 L/min, whereas for the VCCVM, it is 0.81 L/min, resulting in an improvement of approximately 30%.

**Figure 15:**
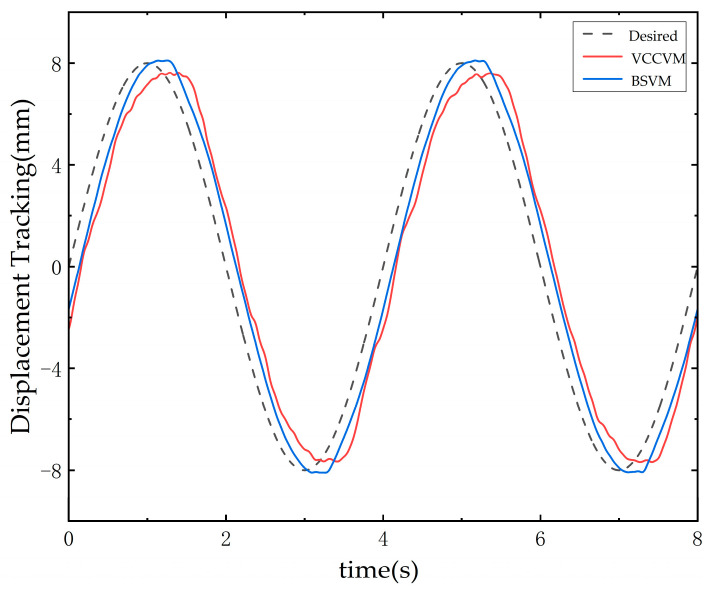
Sine wave signal tracking curve.

**Figure 16:**
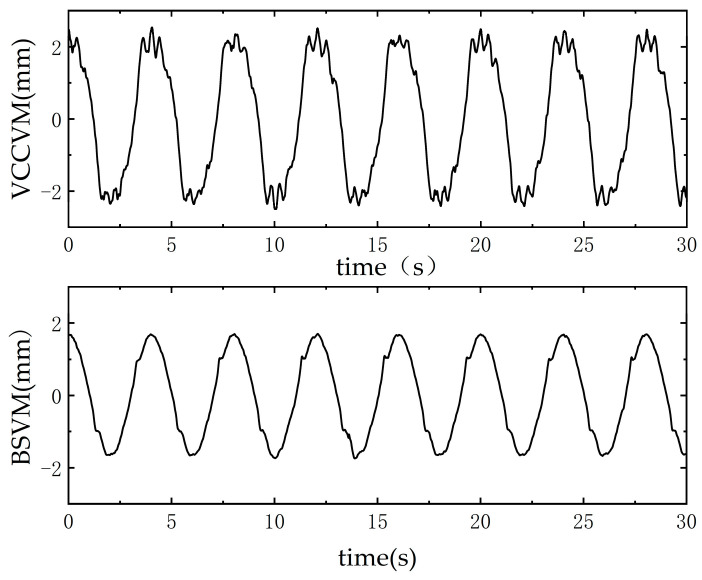
Sinusoidal tracking error.

After changing the input signal to a sine wave with a frequency of 0.25 Hz, it can be observed from Figure 15 that the output signal of BSVM is closer to the displacement command signal compared to VCCVM. The results indicate that the BSVM exhibits a smaller tracking error, with the error converging within 1.24 mm, whereas the tracking error of VCCVM converges within 1.58 mm. Figure 16 presents the error curves for sine displacement tracking. It is evident that the BSVM system exhibits smoother error behavior, smaller deviations, and more consistent tracking performance compared to the VCCVM system, which demonstrates larger fluctuations and greater deviations.

### 4.4. Comparative Experimental Study on the Performance of PID and VFSMC

**Figure 17:**
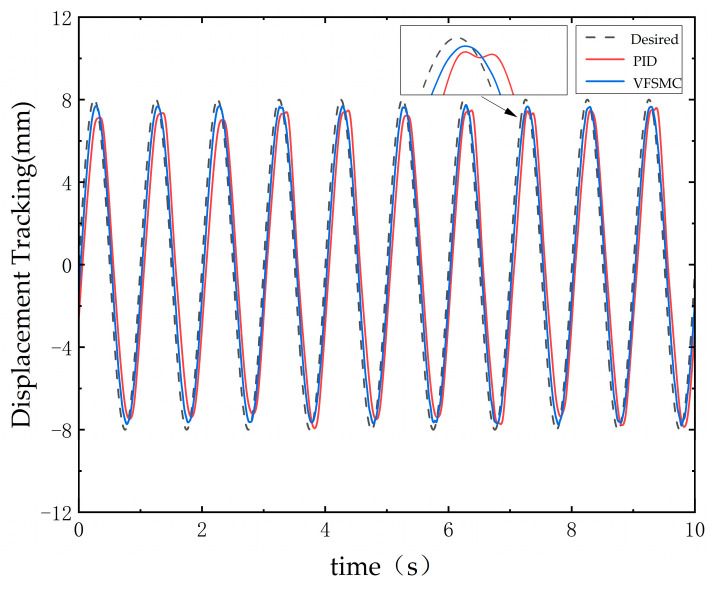
Tracking curves for sine wave signals VFSMC and PID.

The experiment was conducted on the BSVM testing platform with a maximum rotational speed setting of 2000 rpm, where the PID controller and VFSMC were comparatively analyzed. Similarly to Section 4.3, viscous resistance was considered as a disturbance force. The experiment was set up under identical operating conditions and target displacement outputs to compare the proposed control algorithm with the classical PID controller. The tracking performance for a 1 Hz sinusoidal input, as shown in Figure 17, demonstrates that the maximum error of VFSMC was reduced by 38% compared to PID. Furthermore, it can be observed that VFSMC exhibits stronger disturbance rejection capabilities when disturbance forces are introduced.

Future work could explore more precise modeling approaches or adaptive control strategies to enhance the robustness and adaptability of BSVM, thereby improving its tracking performance under complex operating conditions and disturbance effects. Additionally, future research will investigate various improvement methods, such as nonlinear compensation techniques, multi-model control approaches, and disturbance observation and compensation techniques, to further optimize the control performance of BSVM.

## 5. Conclusions

This paper proposes a novel variable mechanism based on a ball screw structure, integrating the structural design of the ball screw with a customized control strategy to achieve precise displacement control. The main contributions of this work are as follows:

A mechanical–hydraulic coupled model is established for the proposed Ball Screw Variable Mechanism (BSVM), and its performance is analyzed. A new control algorithm is developed to enhance the system’s performance. The feasibility and accuracy of the proposed model and algorithm are verified through simulations and experiments. The simulation results validate the effectiveness of the proposed model, while experimental comparisons demonstrate the superiority of the BSVM system and the robustness of the algorithm. Specifically, when the system reaches a stable state, the steady-state error of the BSVM system is ±0.06 mm, compared to ±0.085 mm for the VCCVM system. These experimental results indicate that the BSVM system significantly improves both the accuracy and stability of the system.

## Figures and Tables

**Table 1:** Symbols and descriptions.

Symbols	Descriptions
$Q_{L}$	Output flow rate
$K_{t}$	Flow gain coefficient of the valve
$u_{1}$	Electrical signals of electromagnetic proportional valves
$p_{s}$	Supply pressure of the control oil source
$p_{L}$	Pressure differential between two chambers
A	Cross-sectional area of the cylinder
x	Displacement of variable mechanism
$C_{tc}$	Total leakage coefficient
$V_{t}$	Variable cylinder total volume
$\beta_{e}$	Elastic modulus of the fluid
m	Valve-controlled cylinder mass
c	Viscous damping coefficient of the variable piston cylinder
*k*	System spring stiffness
$F_{L}$	External disturbance
$p_{n}$	External load pressure
β	Tilt angle of the swash plate
L	Distance from the point of action of the variable mechanism on the swash plate to the rotation axis of the swash plate
R	Plunger distribution radius in cylinder bore
$\varphi_{n}$	Rotational angle of the cylinder corresponding to the plunger shoe assembly
N	Number of plungers in the high-pressure discharge region
$m_{z}$	Plunger mass
ω	Spindle speed
E	Elastic modulus of the screw rod
$L_{b}$	Total length of the ball screw
α	Contact angle between the balls and the screw
γ	Helix angle of the screw

## Data Availability

The data presented in this study are available upon request from the corresponding author.
